# Natural bladder filling alters resting brain function at multiple spatial scales: a proof-of-concept MAPP Network Neuroimaging Study

**DOI:** 10.1038/s41598-020-76857-x

**Published:** 2020-11-16

**Authors:** Ishtiaq Mawla, Andrew Schrepf, Eric Ichesco, Steven E. Harte, David J. Klumpp, James W. Griffith, Eric Strachan, Claire C. Yang, Henry Lai, Gerald Andriole, Vincent A. Magnotta, Karl Kreder, Daniel J. Clauw, Richard E. Harris, J. Quentin Clemens, J. Richard Landis, Chris Mullins, Larissa V. Rodriguez, Emeran A. Mayer, Jason J. Kutch

**Affiliations:** 1 Neuroscience Graduate Program, University of Michigan, Ann Arbor, MI USA; 2 Department of Anesthesiology, Chronic Pain and Fatigue Research Center, University of Michigan, Ann Arbor, MI USA; 3 Department of Urology, Feinberg School of Medicine, Northwestern University, Chicago, IL USA; 4 Department of Medical Social Sciences, Feinberg School of Medicine, Northwestern University, Chicago, IL USA; 5 Department of Psychiatry, University of Washington, Seattle, WA USA; 6 Department of Urology, University of Washington, Seattle, WA USA; 7 Department of Anesthesiology, Washington University, St. Louis, MO USA; 8 Division of Urologic Surgery, Department of Surgery, Washington University, St. Louis, MO USA; 9 Department of Radiology, University of Iowa, Iowa City, IA USA; 10 Department of Urology, University of Iowa, Iowa City, IA USA; 11 Department of Urology, University of Michigan, Ann Arbor, MI USA; 12 Department of Biostatistics, Epidemiology and Informatics, Perelman School of Medicine at the University of Pennsylvania, Philadelphia, PA USA; 13 National Institute of Diabetes and Digestive and Kidney Diseases, National Institutes of Health, Bethesda, MD USA; 14 Department of Urology, University of Southern California, Los Angeles, CA USA; 15 G. Oppenheimer Center for Neurobiology of Stress and Resilience, Vatche and Tamar Manoukian Division of Digestive Diseases, David Geffen School of Medicine at the University of California, Los Angeles, CA USA; 16 Division of Biokinesiology and Physical Therapy, University of Southern California, 1540 E. Alcazar Street, CHP 155, Los Angeles, CA 90033 USA

**Keywords:** Functional magnetic resonance imaging, Bladder

## Abstract

Neural circuitry regulating urine storage in humans has been largely inferred from fMRI during urodynamic studies driven by catheter infusion of fluid into the bladder. However, urodynamic testing may be confounded by artificially filling the bladder repeatedly at a high rate and examining associated time-locked changes in fMRI signals. Here we describe and test a more ecologically-valid paradigm to study the brain response to bladder filling by (1) filling the bladder naturally with oral water ingestion, (2) examining resting state fMRI (rs-fMRI) which is more natural since it is not linked with a specific stimulus, and (3) relating rs-fMRI measures to self-report (urinary urge) and physiologic measures (voided volume). To establish appropriate controls and analyses for future clinical studies, here we analyze data collected from healthy individuals (N = 62) as part of the Multidisciplinary Approach to the Study of Chronic Pelvic Pain (MAPP) Research Network. Participants orally ingested approximately 350 mL of water, and had a 10 min “fuller bladder” rs-fMRI scan approximately 1 h later. A second 10 min “empty bladder” rs-fMRI scan was conducted immediately following micturition. We examined multiple spatial scales of brain function, including local activity, circuits, and networks. We found changes in brain function distributed across micturition loci (e.g., subregions of the salience, sensorimotor, and default networks) that were significantly related to the stimulus (volume) and response (urinary urge). Based on our results, this paradigm can be applied in the future to study the neurobiological underpinnings of urologic conditions.

## Introduction

The central nervous system (CNS) communicates with the bladder to signal when micturition should occur^1–3^. The brain monitors for excessive bladder volume, giving rise to the conscious perception of urinary urge, which then motivates the voluntary act of micturition under appropriate circumstances. Aberrant communication between the brain and the bladder may underlie several pathological urologic symptoms, including pain, incontinence, urgency and frequency^4–7^.

Over the past two decades, noninvasive functional brain imaging has shed light on the multiple brain regions involved in regulating the bladder^1,8^. These encompass midbrain and subcortical structures [pontine micturition center, periaqueductal gray (PAG), thalamus] and cortical structures (supplementary motor area, insular cortex, anterior cingulate, prefrontal cortex), which have been reviewed previously^8^. A key conceptual model that has emerged from this work is that the brain contains separate circuits for control of urine storage: frontal cortex circuits that monitor bladder state and evaluate the social appropriateness of voiding^9^, and distinct circuits involving the cingulate and supplementary motor area that engage protective motor mechanisms during the perception of urinary urge^10,11^.

The aforementioned brain regions have been discovered through neuroimaging studies combined with catheter-based urodynamic testing. Typically, the procedure involves multiple trials of rapid infusion of liquid into the bladder through a urinary catheter, while the brain is simultaneously scanned using functional Magnetic Resonance Imaging (fMRI)^6,12^. The difference in the Blood Oxygenated Level Dependent (BOLD) signal in the brain between phases of bladder activity (e.g., filling vs. voiding) is then used to make inferences about the neural mechanisms of bladder function. Although urodynamic testing in fMRI has an advantage of improved experimental control over bladder volume, it has several limitations. First, this procedure fills the bladder at supraphysiologic rates through an artificial mechanism; therefore, the brain response reported in these studies may be partially driven by salience, attention, patient discomfort and anxiety, and other factors that are beyond actual bladder activity. Second, certain patient populations (e.g. those with pelvic pain) may be averse to the catheter procedure, limiting sample sizes. Finally, brain activity in urodynamic studies is examined time-locked to the infusion of fluid into the bladder; it is more natural to examine function in the brain without other specific stimuli (resting-state) when the bladder is empty compared to when it is not.

In response to the aforementioned limitations and challenges, the Multidisciplinary Approach to the Study of Chronic Pelvic Pain (MAPP) research network proposed to create a standardized multi-site natural bladder filling paradigm with resting-state fMRI (rs-fMRI) as part of the network’s second phase (MAPP-II) clinical protocol, the Trans-MAPP Symptom Patterns Study (SPS). The primary purpose of the MAPP-II SPS was to longitudinally track symptoms and underlying biological factors in patients with Urologic Chronic Pelvic Pain Syndrome (UCPPS), though data were collected from healthy controls as well for cross-sectional comparison. The MAPP-II bladder filling paradigm needed to (1) be tolerable to patients with UCPPS, (2) evoke an urge to urinate and a brain response in healthy individuals to provide suitable controls, and (3) had to provide assessments of pain, urinary urge, and voided volume at critical time points.

The need for such a standardized acquisition and analysis protocol is pressing. A few previous studies have examined natural bladder filling or/and rs-fMRI data in UCPPS patients^4^, other urologic conditions^13^, and healthy controls^9,14^, but none have designed and implemented a comprehensive multi-site paradigm to examine the relation between self-reported experiences (pain and urinary urge), physiologic measures (bladder volume), and multi-scale rs-fMRI analysis.

In the current study, we examine several key features of the MAPP-II bladder filling paradigm in the MAPP-II healthy control cohort at the baseline (first) visit to provide the groundwork for future MAPP-II studies in UCPPS patients and future clinical studies in other patient populations using a similar bladder filling paradigm. In this study, we address the proportion of participants in whom the paradigm induced urinary urge (the response rate), the association between self-report (pain and urinary urge) and physiologic (voided volume) measures, and finally examine three rs-fMRI analysis techniques, each operating at a different spatial scale, to quantify brain response to natural bladder filling.

## Methods

### Participants

Participants who were free of pain and urologic symptoms (N = 62:33 F 29 M) were recruited to serve as controls as part of the second phase of the NIDDK-funded Multidisciplinary Approach to the Study of Chronic Pelvic Pain (MAPP) Research Network (MAPP-II). Participant recruitment took place at six sites across the United States: Northwestern University (NU), Chicago, Illinois (N = 10), University of California/University of Southern California, Los Angeles (UCLA/USC) (N = 11), University of Iowa (UI), Iowa City (N = 10), University of Michigan (UM), Ann Arbor (N = 11), University of Washington (UW), Seattle (N = 8), and Washington University (WashU), St. Louis, Missouri (N = 12). All study protocols were approved by the Institutional Review Boards at NU, UCLA/USC, UI, UM, UW, and WashU. All study protocols were followed according to the Declaration of Helsinki. All participants provided informed consent for participating in the study.

### Inclusion and exclusion criteria

Inclusion and exclusion criteria have been previously published^15^. Participant inclusion criteria included: (1) greater than or equal to 18 years of age, (2) ability to speak, read, and understand English, (3) zero on a pain, pressure or discomfort scale, (4) no chronic pain in the pelvic or bladder region, and no reports of chronic pain in any other body region, and (5) no urological symptoms. In addition, participant exclusion criteria included: (1) on-going symptomatic urethral stricture, (2) on-going neurological disease or disorder affecting the bladder or bowel fistula, (3) history of cystitis caused by tuberculosis, radiation therapy or Cytoxan/cyclophosphamide therapy, (4) augmentation cystoplasty or cystectomy, (5) active autoimmune or infectious disorder (such as Crohn’s Disease or ulcerative colitis, lupus, rheumatoid arthritis, multiple sclerosis, or HIV), (6) history of cancer (with the exception of skin cancer), (7) current major psychiatric disorder or other psychiatric or medical issues that would interfere with study participation (e.g. dementia, psychosis, upcoming major surgery, etc.), (8) severe cardiac, pulmonary, renal, or hepatic disease that in the judgment of the study physician would preclude participation in this study, (9) definitive treatment for acute epididymitis, urethritis, vaginitis, (10) history of unevaluated hematuria, (11) history of cystoscopy with hydrodistention or kenalog injection, and (12) a positive urine culture. In addition, males were excluded based on (13) diagnosis of unilateral orchalgia, without pelvic symptoms, (14) history of transurethral microwave thermotherapy (TUMT), transurethral needle ablation (TUNA), balloon dilation, prostate cryo-surgery, or laser procedure, and (15) prostate biopsy or transurethral resection of the prostate (TURP) conducted within the last three months. Moreover, females were excluded if they had (16) a positive pregnancy test.

### Study design

The overall design of the MAPP-II SPS for control participants, which is abbreviated compared to that for the UCPPS cohort, includes screening and enrollment of eligible participants during an in-clinic screening/eligibility visit (Week 0) during which participants were asked to provide biological samples, undergo a physical and pelvic exam, complete phenotyping questionnaires, and participate in the bladder filling and neuroimaging protocol (described below), as well as a quantitative sensory testing (QST) protocol. After the Week 0 visit, eligible participants were asked to complete a series of online questionnaires 3 months after the initial visit and asked to come back in 6 months for a follow-up and final study visit. The 6-month visit was identical to the baseline visit. This manuscript only focuses on results from the neuroimaging protocol at baseline.

### MAPP-II bladder filling and neuroimaging protocol

**Figure 1:**
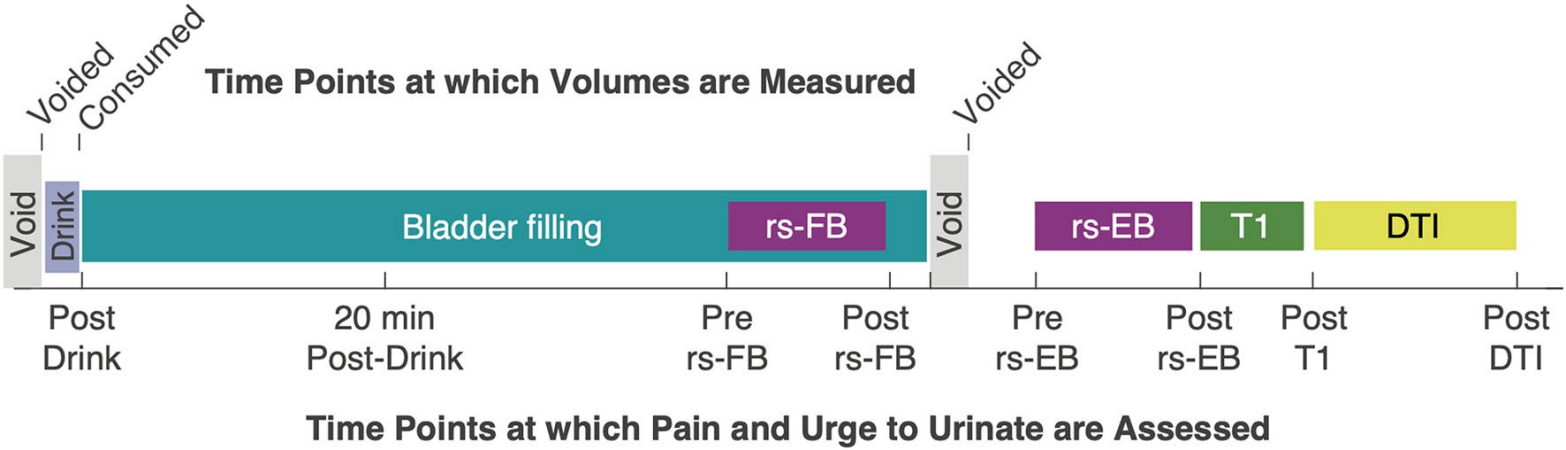
MAPP-II bladder filling and neuroimaging protocol. Participants voided and then consumed approximately 350 mL of water approximately 40 min prior to entering the MRI scanner. A resting-state fMRI (rs-fMRI) scan was first acquired with fuller-bladder (rs-FB). Participants then exited the scanner and fully voided the bladder through natural urination. Participants then returned to the scanner and another resting state scan was acquired with empty bladder (rs-EB). These resting state scans were followed by structural scans: T1-weighted and diffusion tensor imaging (DTI). Tick marks along the top show time points at which volumes (either consumed or voided) were measured. Tick marks along the bottom show time points at which participants rated pain and urge to urinate (separately) on 0–10 scales.

The MAPP-II bladder filling and neuroimaging protocol is shown in Fig. 1. This study used a natural bladder filling paradigm based on oral ingestion of water to create two states of urinary urge in each individual—a “fuller” bladder (FB) state with higher urinary urge and an empty bladder (EB) state with lower urinary urge. At specific time points throughout the procedure described below, the experimenter obtained verbal ratings of pain (0–10 scale) and urge to urinate (0–10 scale). Upon arrival to the neuroimaging session, participants first fully voided their bladder. The volume voided was recorded using a graded specimen collection hat. Following the initial void, participants drank 12 oz (~ 350 mL) of bottled water. The start time, end time, and actual volume of water ingested was recorded. Participants then waited for approximately 40 min before going inside the MRI scanner. During this wait time, participants relaxed, changed into MRI safe attire, and filled out questionnaires. At a fuller-bladder state, participants were positioned in the MRI scanner and were instructed to stay still and awake with their eyes open. At this point, a 10-min resting state fMRI (rs-fMRI) was recorded with a fuller bladder (rs-FB). Following rs-FB, the participant left the scanner and was instructed to void fully into the urine collection hat, and the volume voided was again measured. This void volume was used as a proxy measure for the bladder volume during rs-FB. The participant then entered the scanner again and repeated another rs-fMRI scan in the empty bladder state (rs-EB). Following rs-EB, T1-weighted and Diffusion Tensor Imaging (DTI) scans were acquired. This manuscript will focus on the results from rs-FB and rs-EB, which are the most relevant to natural bladder filling. The neuroimaging protocol described was identical for control participants and the UCPPS cohort, although the UCPPS cohort repeated this full procedure (shown in Fig. 1) at additional time points.

### Definition of responders to bladder filling

Measures of urge to urinate before and after the rs-FB and rs-EB scans were averaged to create one urinary urge score associated with each scan. We defined “responders” to the bladder filling protocol primarily by those with higher urinary urge during rs-FB compared to rs-EB. However, we also included a small number of participants as responders if they had greater than 0 urinary urge during rs-FB even if urinary urge did not change between rs-FB and rs-EB, since the brain in these individuals could reasonably be assumed to be processing urge information, and lack of change in urinary urge might be corroborated by lack of change in brain activity. To further clarify, a hypothetical participant could have a rs-FB urge score of *x*, rs-EB urge score of *x*, and the resulting change of 0. Despite the change value being 0, this participant would be included as a responder because the bladder provocation elicits an non-zero urge rating of *x* at rs-FB. “Non-responders” had both no change in urinary urge between rs-FB and rs-EB, and no urinary urge at rs-FB. The difference in urge (i.e. rs-FB minus rs-EB) was used for whole-brain regression analyses in responders. Difference in urge could not be used for whole-brain regression as the values were 0 (see group analysis section below).

### MRI scanning

The Neuroimaging Core of the G. Oppenheimer Center for Neurobiology of Stress and Resilience (CNSR) at UCLA operated as the hub for neuroimaging operations in MAPP-II. The scanning parameters for rs-fMRI, T1, and DTI imaging have been described as part of the previous MAPP-I study^16^. UCLA-CNSR took several steps throughout the MAPP-II data acquisition period to ensure multi-site MRI quality. Before start of recruitment, UCLA-CNSR obtained qualification scans of a healthy individual from each site to check scanning parameter compliance, as well as obtaining scans from a “travelling human phantom” (the same individual scanned at all 6 sites with all sequences but without the bladder filling protocol). Throughout the study, scans from all sites were uploaded to the UCLA-CNSR repository where they were promptly reviewed for parameter compliance and image quality.

Here we briefly describe the rs-fMRI and T1 parameters. rs-fMRI scans were acquired with a single shot echo planar imaging (EPI) pulse sequences with conventional rectangular Cartesian sampling. Basic pulse sequence parameters were as follows TR = 2000 ms, TE = 30 ms, Flip angle = 77°, FoV = 220 mm × 220 mm, Resolution = 64 × 64, Phase encode direction = A > P, Slice thickness = 4.0 mm, Slice gap = 0.5 mm, Slice acquisition = ascending (not interleaved), Slices per volume = 34–40 to cover entire brain, Phased array acceleration factor = 2, Bandwidth = maximum to accommodate resolution specifications, Orientation = axial-oblique parallel to the line between the anterior and posterior commissures, Number of volumes = 300 (10 min acquisition).

The MP-RAGE pulse sequence was used for high resolution T1-weighted 3D volume imaging. The equivalent pulse sequence on a GE scanner was the 3D FSPGR IR. Basic parameters were as follows: TR = 2300 ms, TE = 2.98 ms, TI = 900 ms, Flip angle = GE: 11°, Siemens: 9°, Philips: 9°, FoV = 256 mm × 256 mm, Resolution = 256 × 256, Slices per volume = 240 (or maximum available while maintaining all other parameters), Slice thickness = 1 mm, Inversion = Slice Selective, parallel imaging acceleration factor = 2, Phase encode direction = left–right and superior-inferior, Orientation = axial-oblique parallel to the line between the anterior and posterior commissures.

### Preprocessing

All neuroimaging preprocessing was performed using fMRIprep 1.1.8^17^, which utilizes the Nipype 1.1.3 platform^18^. The T1-weighted (T1w) image underwent intensity correction using N4BiasFieldCorrection (ANTs 2.2.0)^19^ and skull-stripping using antsBrainExtraction.sh (ANTs 2.2.0). Surface reconstruction was performed with recon-all (FreeSurfer 6.0.1)^20^, with additional refinement of the brainmask^21^. Nonlinear registration and spatial normalization to the 2009c ICBM152 template was performed with antsRegistration (ANTs 2.2.0)^22^. Tissue segmentation of cerebrospinal fluid (CSF), white-matter (WM), and gray-matter (GM) was performed on the brain-extracted T1w using fast (FSL 5.0.9)^23^.

rs-FB and rs-EB images also underwent fMRIprep preprocessing. Reference images were co-registered with 9 degrees-of-freedom to the T1w reference using boundary-based registration (bbregister, FreeSurfer)^24^. Head-motion realignment was performed using mcflirt (FSL 5.0.9)^25^. Images were warped to MNI152NLin2009cAsym standard space and resampled to 2 × 2 × 2 mm voxel dimension to allow for cross-subject comparison. Framewise Displacement (FD) was calculated using Nipype^26^. Six physiological regressors were extracted for principal component-based noise correction based on anatomical CSF and WM masks computed in native space (aCompCor)^27^. Following the fMRIprep minimally preprocessed pipeline, the preproc.nii images were skull-stripped using a dilated MNI mask (fslmaths). Based on recent recommendations^28^, six head motion parameters from mcflirt, aforementioned six aCompCor regressors, and high-pass temporal filtering (0.01 Hz) were done simultaneously using 3dTproject function in AFNI. Finally, 3DBlurToFWHM was used to estimate smoothness of each image followed by iterative smoothing until the images reached a target smoothness of 6 mm FWHM.

### Motion assessment

A conservative criterion [3 mm maximum Framewise Displacement (max-FD), as opposed to the recent recommendation of 5 mm]^29^ was used to exclude high motion runs to ensure the results are not confounded by motion artifacts. Out of 124 rs-FB and rs-EB runs collected from participants, 9 runs had a max-FD above 3 mm and were not included for further analysis. For this analysis, each participant required a pair of rs-FB and rs-EB for group analysis.

### Assessment of local activity: slow-5 fractional amplitude of low frequency fluctuation (fALFF)

We first assessed local activity in discrete brain regions using Amplitude of Low Frequency Fluctuations (ALFF). ALFF is a local measure of spontaneous brain activity, which is computed from the power spectrum of the BOLD signal. Fractional ALFF (fALFF) accounts for physiological confounds and individual differences by examining power in the frequency range of interest compared to power in the total frequency range. Here we focused on slow-5 oscillations (0.01–0.027 Hz), as we have previously identified changes specific to slow-5 fALFF in urologic disease compared to healthy controls^30^, and have correspondingly shown that slow-5 fALFF may predictably respond to different repetitive transcranial magnetic stimulation (rTMS) sequences known to both increase and decrease neural activity neuromodulation of BOLD activity in urologic conditions^31^. In order to generate slow-5 fALFF images, the 3dRSFC AFNI function was used instead of the 3dTproject algorithm above, with a bandpass filter set to 0.01–0.027 Hz. The other steps were kept the same. Slow-5 fALFF images were z-standardized by subtraction of the global image mean and division by the global standard deviation.

### Assessment of circuits: seed connectivity of the periaqueductal gray

Circuits (i.e. communication between brain regions), can be assessed through connectivity of discrete seed regions. Here we chose a seed region in the PAG since this region has been an established hub in the brain networks controlling urine storage and voiding^8^. Seed-to-voxel correlation analysis was used to evaluate whole-brain connectivity maps for the PAG. This PAG seed was defined as a 3 mm radius sphere from a comprehensive meta-analysis of the PAG from previous bladder control studies (centroid MNI coordinates x = 1, y = − 25, z = − 12, Fig. 4A)^32^. Averaged fMRI signal from this PAG seed (fslmeants) was used as a GLM regressor for each individual (fsl_glm) to obtain parameter estimates and associated variances. These parameter estimates were then passed on to group level analysis.

### Assessment of networks: default mode, salience, and sensorimotor subsystems

Networks were assessed with Independent Components Analysis (ICA), a spatiotemporal decomposition method to extract the major networks of the brain. ICA was performed on FSL MELODIC on temporally concatenated rs-FB and rs-EB data, with a dimensionality constraint of 25 Independent Components (ICs). The constraint of 25 ICs was in accordance with previous studies where two resting state runs per individual were concatenated^33,34^. Here, we focused on three major networks involved in bladder control^8^: salience network (SLN, comprised of the anterior cingulate and the anterior insula, involved in encoding of salient stimuli), default network (DMN, comprised of medial prefrontal and posterior cingulate, involved in interoception), and the sensorimotor network (SMN, comprised of the sensory, motor, and supplementary motor areas, involved in motor and sensory responses). Best fit ICs for the SLN, DMN, and SMN were found through spatial correlation with the Yeo 7-network template^35^. Dual regression was used to explore whole-brain connectivity of each network and resultant connectivity parameter estimate and variance maps for each individual were passed up to group-level analyses.

### Group analysis for associations with urinary urge and void volume

For analyses at each spatial scale, the resultant images for each individual (i.e. z-standardized slow-5 fALFF, parameter estimates for PAG connectivity, and parameter estimates for SLN, DMN, and SMN connectivity, respectively) were passed up to group-level difference analyses. The FSL Local Analysis of Mixed Effects^36^ (FLAME1 + 2) was used to improve mixed-effects variance estimation. We performed whole-brain linear regression analyses to assess the association between images at each spatial scale and measures of bladder perception (i.e. urinary urge) and bladder stimulus (i.e. void volume) at the time of the scan. All regressions were conducted in responders only, as we did not hypothesize brain activity to be highly modulated in non-responders. Non-responders were not used for further analyses. For these analyses in responders, the rs-FB minus rs-EB parameter estimate difference map was calculated for each subject and input along with regressors of demeaned urinary urge and separately, void volume. Age and sex were included as regressors of no interest. Multiple comparisons correction was conducted using gaussian random field (GRF) cluster threshold (Z > 2.3) and significance at p < 0.05.

## Results

### Participant demographics and characteristics

Basic MAPP-II control data are presented in Table 1. A total of 58 of the 62 participants passed neuroimaging quality controls (QC). The overall response rate was 63% (37 of 58) in this healthy population. Responders (17 M, 20 F) did not differ by sex from the non-responders (10 M, 11 F). Responders and nonresponders did not differ in either the amount of urine voided at the beginning of the protocol, or in the amount of water consumed. Responders were significantly younger than the non-responders, and also voided a significantly greater volume of urine after the rs-FB scan. Across both responders and non-responders, the correlation value of void volume and age is − 0.09 (Pearson’s r, p = 0.49). The correlation is also not significant within responders (r = − 0.06, p = 0.74) or non-responders (r = 0.28, p = 0.21), and the two correlation values do not significantly differ from each other (Fisher’s z = − 1.19, p = 0.23). While the number of participants at each site was low, 5 of 6 sites had a majority of responders while the 6th site had a 42% response rate (5 of 12). One responder had an rs-FB rating of 1, rs-EB rating of 1, and hence the change was zero; this responder was used for further analysis as evoked urge at rs-FB was a non-zero value.

**Table 1:** Participant demographics and volumes consumed and voided.

N = 58 of 62 passed neuroimaging QC	N = 37 responders	N = 21 non-responders	p-value
Sex	17 M 20 F	10 M 11 F	0.9022
Age (years)	37.71 ± 13.93	49.67 ± 13.29	0.0023
Pre-drink void volume (mL)	112 ± 90	83 ± 68	0.2130
Drink consumed volume (mL)	354 ± 7	353 ± 3	0.2740
Post-rs-FB void volume (mL)	283 ± 183	182 ± 110	0.0250
**Number of participants by study site**			
NU	6	2	
USC/UCLA	6	5	
UI	7	3	
UM	8	2	
UW	5	2	
WashU	5	7	

### Effects of natural bladder filling on urinary urge in responders

**Figure 2:**
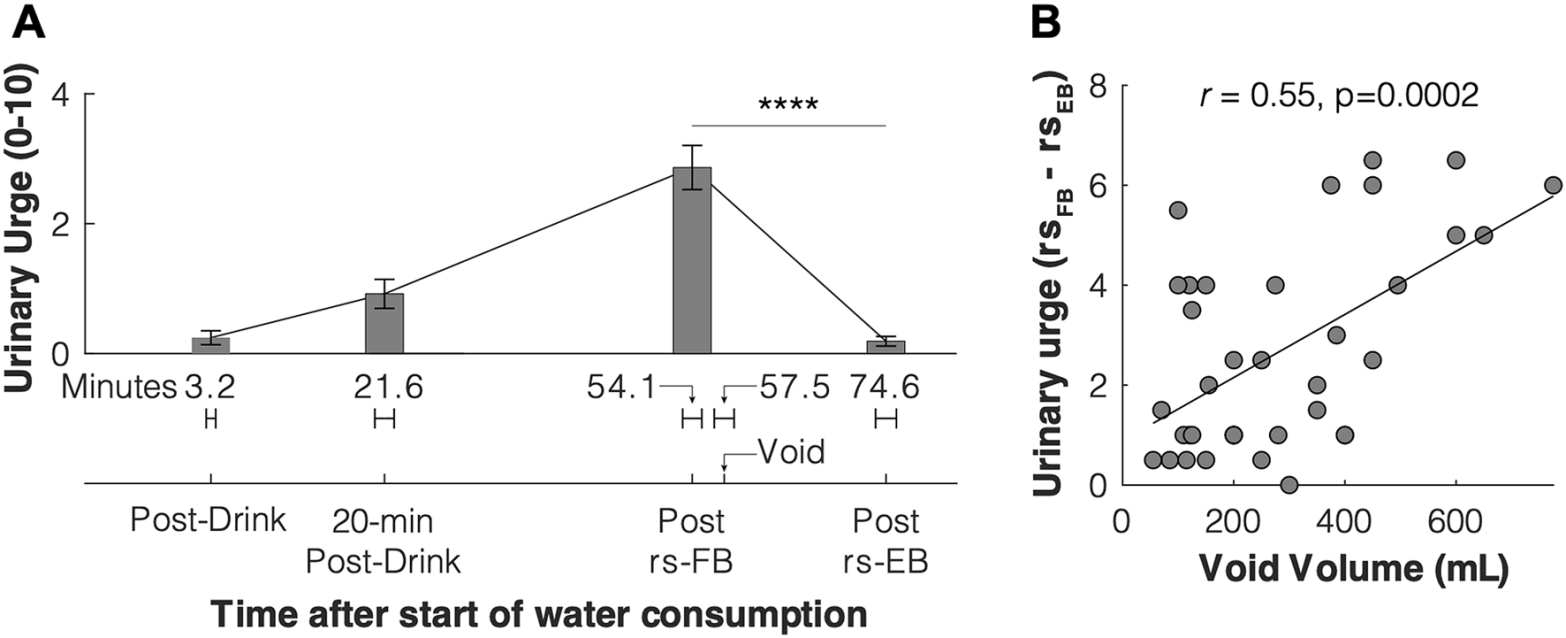
(**A**) Change in urinary urge across the protocol for the “responders”. Ticks on x-axis represent average times in the protocol: when water consumption was finished (post-drink), approximately 20 min later (20 min post-drink), after the fuller bladder resting state scan (rs-FB), when the participant voided (Void), and after the empty bladder resting state scan (rs-EB). Horizontal error bars represent standard error of the mean for the indicated times, and the average time in minutes is shown for each timepoint. Vertical axis represents the participant’s self-reported urinary urge at the different time points. Vertical error bars represent the standard error of the mean. Urinary urge values shown at the post rs-FB and post rs-EB time points are the average of pre and post urinary urge values reported by participants for that scan. Paired t-test between rs-FB and rs-EB urinary urge shows a significantly greater urinary urge during the rs-FB scan (p < 0.0001). (**B**) Relation between bladder volume and urinary urge change. Voided volume after rs-FB was taken as a surrogate for approximate bladder volume during rs-FB. Increases in voided volume were significantly correlated with the difference in urinary urge between rs-FB and rs-EB (p = 0.0002).

The MAPP bladder filling protocol produced changes in urinary urge related to bladder volume (Fig. 2). Urinary urge was minimal when first assessed after voiding and drinking water at the beginning of the protocol at the “Post-Drink” time-points (Fig. 2A). Urinary urge was observed to be rising when assessed approximately 20 min after the water consumption at the “20-min Post-Drink” time point (Fig. 2A). Urinary urge reached a maximum during the rs-FB scan, which was approximately 54 min on average after the water consumption (Fig. 2A). After voiding, the participants had the rs-EB scan, which was again associated with minimal urinary urge (Fig. 2A). We also found a significant correlation between the difference in urinary urge between the rs-FB and rs-EB scans and the volume voided after the rs-FB scan, which we interpret as a surrogate for the volume of fluid in the bladder during the rs-FB scan (Fig. 2B).

### Increased bladder volume and urinary urge is related to increased local brain activity

**Figure 3:**
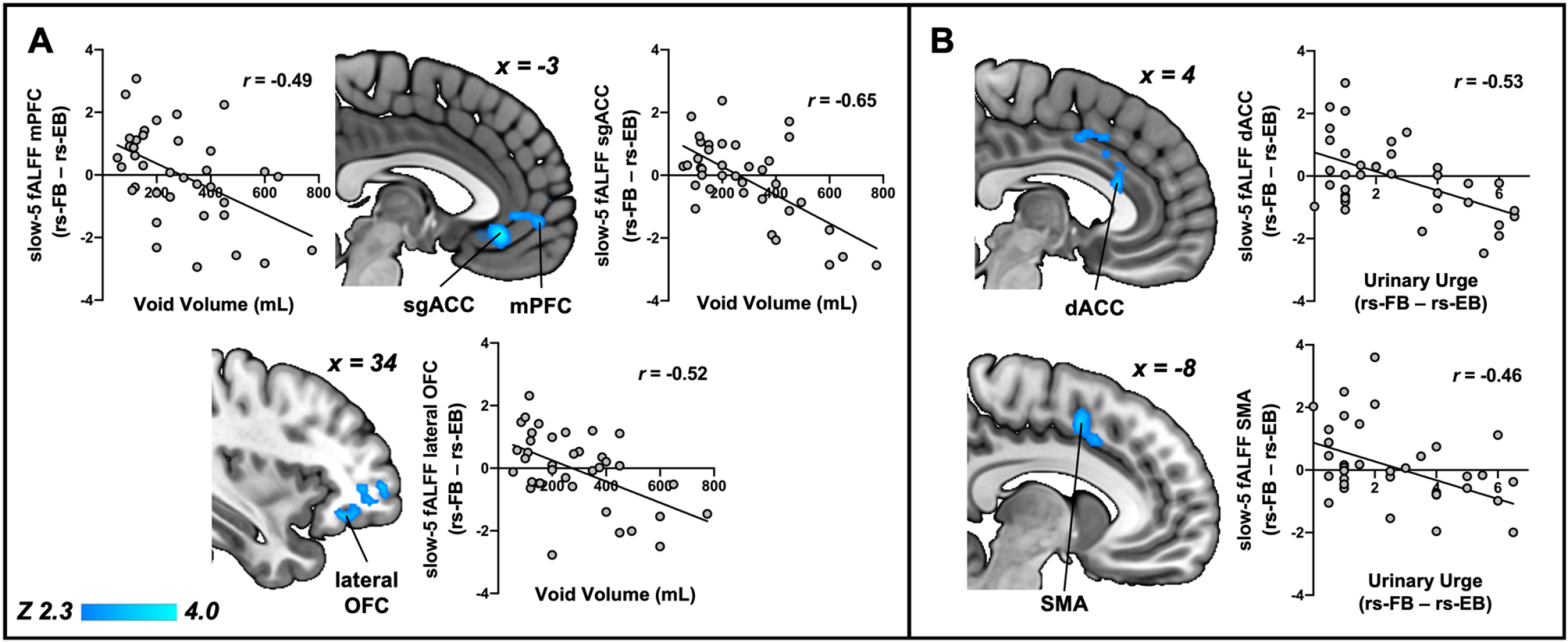
Local brain activity is related to changes in urinary urge and void volume. (**A**) Whole-brain regression of change in slow-5 fALFF (rs-FB minus rs-EB) with void volume showed significant negative associations in the medial Prefrontal Cortex (mPFC), subgenual Anterior Cingulate Cortex (sgACC), and lateral Orbitofrontal Cortex (OFC), such that increases in volume were associated with decreases in fALFF (i.e. increases in local neural activity) in these regions. (**B**) Similarly, whole-brain regression of change in slow-5 fALFF (rs-FB minus rs-EB) with change in urinary urge (rs-FB minus rs-EB) showed significant negative associations in the dorsal Anterior Cingulate Cortex (dACC) and Supplementary Motor Area (SMA). such that increases in urinary urge were associated with decreases in fALFF (i.e. increases in local neural activity) in these regions. All results have been corrected for multiple comparisons (p < 0.05).

This analysis was focused on discovering loci in the brain that encoded stimulus (i.e. voided volume) and resultant perception (i.e. urinary urge). A whole-brain linear regression of the change in fALFF from rs-FB to rs-EB with void volume revealed significant clusters (p < 0.05) that encompassed the subgenual Anterior Cingulate Cortex (sgACC), medial Prefrontal Cortex (mPFC), and lateral Orbitofrontal Cortex (lOFC) (Fig. 3A). In addition, a whole-brain linear regression of the change in fALFF from rs-FB to rs-EB with change in ratings of urinary urge from rs-FB to rs-EB revealed significant clusters (p < 0.05) that encompassed the dorsal Anterior Cingulate Cortex (dACC) and the Supplementary Motor Area (SMA) (Fig. 3B). All these associations were in the negative direction—as urinary urge and voided volume increase, slow-5 fALFF decreases (lower slow-5 fALFF values indicate higher neural activity^31^). Table 2 summarizes these clusters.

**Table 2:** Summary of clusters obtained from whole-brain regression analyses with urinary urge and void.

Cluster size (# voxels)	Cluster *p*-value	Peak				Anatomical location
		*Z*-score	*x* (mm)	*y* (mm)	*z* (mm)	
**Void volume and slow-5 fALFF**						
983	4.0 × 10^–10^	4.31	− 2	34	− 10	Subgenual anterior cingulate (sgACC)
		3.51	34	36	− 14	Lateral orbitofrontal (lOFC)
		3.02	− 3	53	− 4	Medial prefrontal (mPFC)
**Urinary urge and slow-5 fALFF**						
320	6.9 × 10^–4^	3.65	4	24	18	Dorsal anterior cingulate (dACC)
		3.35	− 8	6	50	Supplementary motor area (SMA)
**Void volume and PAG connectivity**						
246	2.7 × 10^–3^	4.03	4	− 8	36	Mid cingulate (MCC)
198	1.3 × 10^–2^	3.61	0	− 36	44	Posterior cingulate (PCC)
191	1.6 × 10^–2^	3.25	− 40	41	28	Dorsolateral prefrontal (DLPFC)
**Urinary urge and PAG connectivity**						
283	7.8 × 10^–4^	3.84	− 2	2	54	Supplementary motor area (SMA)
		3.68	− 2	− 4	40	Mid cingulate (MCC)
236	3.6 × 10^–3^	3.99	38	12	12	Anterior insula (aINS)
		3.53	52	10	18	Inferior frontal gyrus (IFG)
**Void volume and salience network (SLN) connectivity**						
275	9.7 × 10^–6^	4.16	− 2	− 66	− 40	Cerebellar vermis
127	1.2 × 10^–2^	4.04	− 54	− 18	12	Secondary somatosensory cortex (SII)
		3.42	− 36	− 8	16	Posterior insula (pINS)
**Void volume with default mode network (DMN) connectivity**						
114	2.9 × 10^–2^	3.80	64	− 32	42	Inferior parietal lobule (IPL)
**Urinary urge with default mode network (DMN) connectivity**						
121	2.0 × 10^–2^	4.01	16	− 8	76	Primary motor (M1)
		3.43	2	− 8	70	Supplementary motor area (SMA)
112	3.3 × 10^–2^	3.50	42	− 34	38	Inferior parietal sulcus (IPS)
**Void volume with sensorimotor network (SMN) connectivity**						
199	4.3 × 10^–4^	3.48	46	− 44	40	Inferior parietal lobule (IPL)
		3.40	56	− 44	22	Temporoparietal junction (TPJ)
135	1.0 × 10^–2^	3.55	2	44	− 8	Subgenual anterior cingulate cortex (sgACC)/ventromedial prefrontal cortex (vmPFC)
129	1.4 × 10^–2^	4.02	− 8	− 30	− 30	Dorsal pons

### Increased bladder volume and urinary urge is related to increased connectivity of the PAG

**Figure 4:**
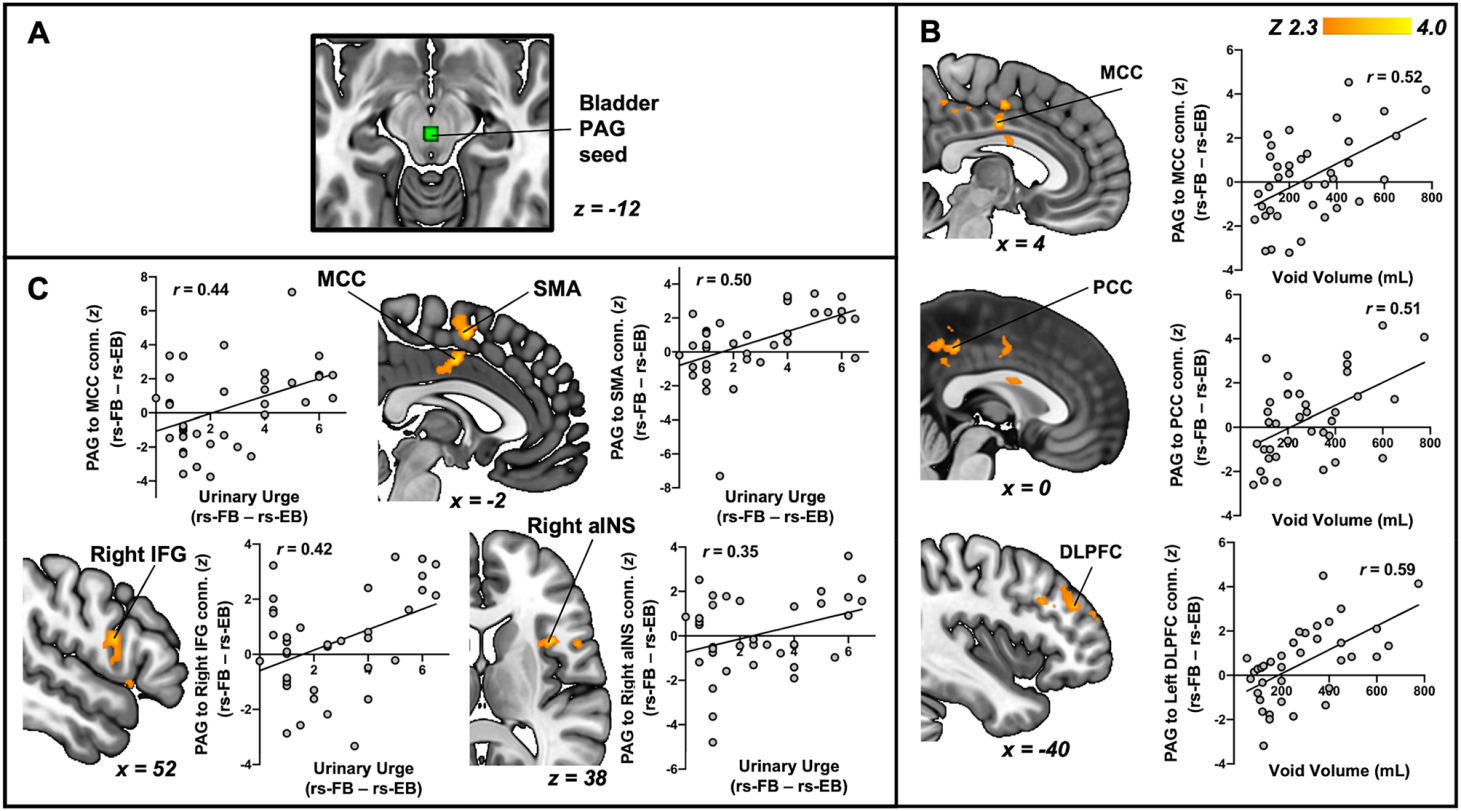
PAG connectivity is related to changes in urinary urge and void volume. (**A**) Bladder PAG seed was defined as a sphere with 3 mm radius with the centroid MNI coordinates x = 1, y = − 25, z = − 12. (**B**) Whole-brain regression of change in PAG connectivity (rs-FB minus rs-EB) with void volume demonstrated positive associations in the Mid-Cingulate Cortex (MCC), Posterior Cingulate Cortex (PCC), and Dorsolateral Prefrontal Cortex (DLPFC), such that increases in void volume were associated with increases in PAG connectivity to these brain regions. (**C**) Whole-brain regression of change in PAG connectivity (rs-FB minus rs-EB) with change in urinary urge (rs-FB minus rs-EB) demonstrated positive associations in the Supplementary Motor Area (SMA), Mid-Cingulate Cortex (MCC), Right Inferior Frontal Gyrus (IFG), and Right anterior Insula (aINS), such that increases in urinary urge were associated with increases in PAG connectivity to these brain regions. All results have been corrected for multiple comparisons (p < 0.05).

Similar to local activity, here we were interested in PAG circuits that encoded stimulus and perception. A whole-brain linear regression of the change in PAG connectivity from rs-FB to rs-EB with void volume revealed significant clusters (p < 0.05) that encompassed the Mid-Cingulate Cortex (MCC), Posterior Cingulate Cortex (PCC), and Dorsolateral Prefrontal Cortex (DLPFC) (Fig. 4B). In addition, a whole-brain linear regression of the change in PAG connectivity from rs-FB to rs-EB with urinary urge revealed significant clusters (p < 0.05) that encompassed the MCC, SMA, right Inferior Frontal Gyrus (IFG), and right anterior Insular Cortex (right aINS) (Fig. 4C). Table 2 summarizes these clusters.

### Increased bladder volume and urinary urge is related to increased salience, sensorimotor, and default network connectivity

**Figure 5:**
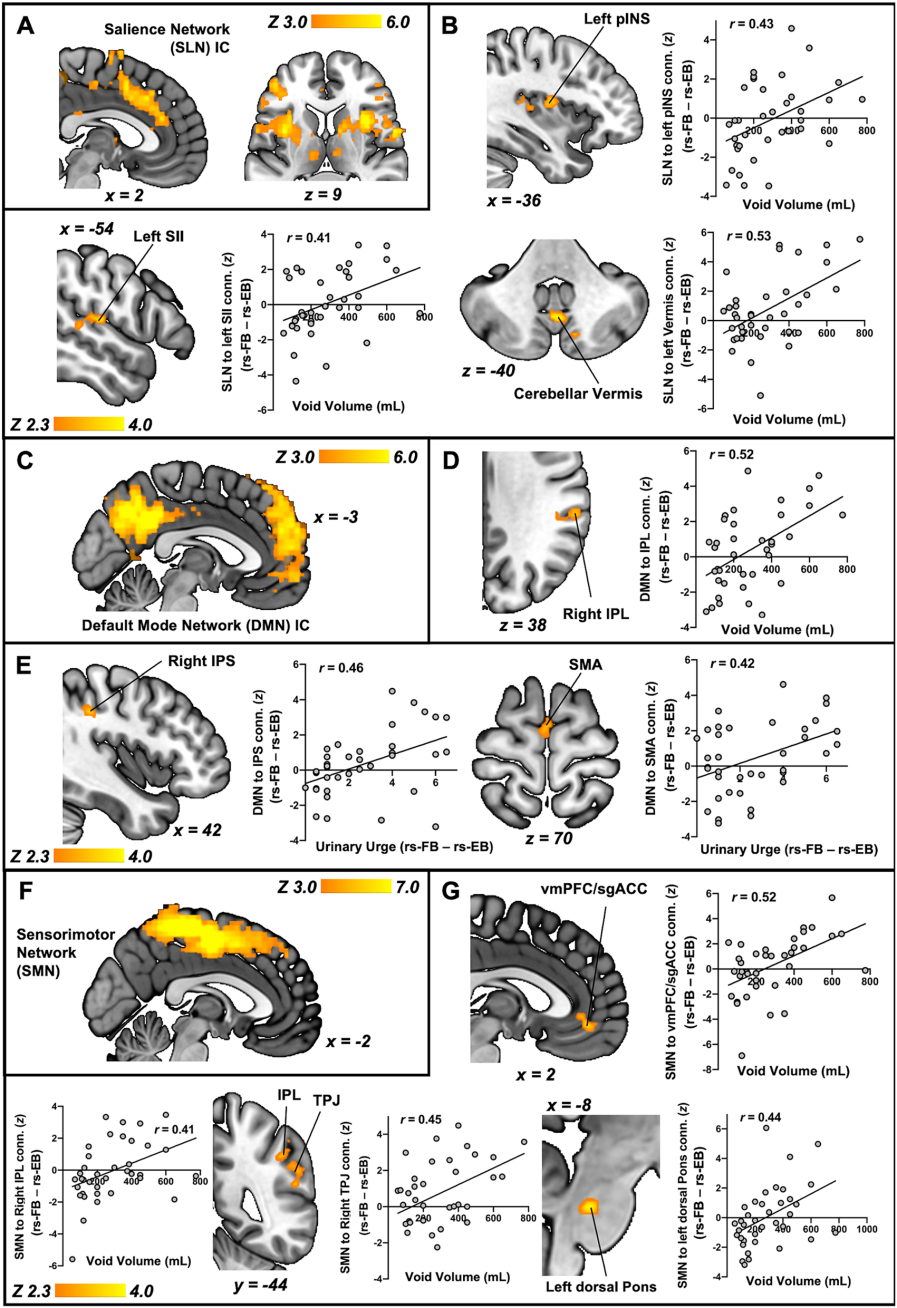
Network connectivity is related to changes in urinary urge and void volume. (**A**) Salience Network (SLN) Independent Component (IC) encompassing the Anterior Cingulate and Insular cortices. (**B**) Whole-brain regression of change in SLN connectivity (rs-FB minus rs-EB) with void volume demonstrated positive associations in the left posterior Insula (pINS), left Secondary Somatosensory cortex (SII), and the Cerebellar Vermis, such that increased volume was associated with increased connectivity of the SLN to these regions. (**C**) Default Mode Network (DMN) IC encompassing the Medial Prefrontal and the Posterior Cingulate cortices. (**D**) Whole-brain regression of change in DMN connectivity (rs-FB minus rs-EB) with void volume demonstrated positive associations in the right Inferior Parietal Lobule (IPL) such that increased volume was associated with increased connectivity of the DMN to this region. (**E**) Whole-brain regression of change in DMN connectivity (rs-FB minus rs-EB) with change in urinary urge (rs-FB minus rs-EB) demonstrated positive associations in the right Inferior Parietal Sulcus (IPS) and the Supplementary Motor Area (SMA) such that increased urinary urge was associated with increased connectivity of the DMN to these regions. (**F**) Sensorimotor Network (SMN) IC encompassing the Primary Somatosensory, Primary Motor, and Supplementary Motor Areas. (**G**) Whole-brain regression of the change in SMN connectivity (rs-FB minus rs-EB) with void volume demonstrated positive associations in the ventromedial Prefrontal Cortex (vmPFC)/subgenual Anterior Cingulate Cortex (sgACC), right Inferior Parietal Lobule (IPL)/Temporoparietal Junction (TPJ), and left dorsal Pons, such that increased volume was associated with increased connectivity of the SMN to these regions. All results have been corrected for multiple comparisons (p < 0.05).

Finally, we assessed the relationship of stimulus and percept with SLN, DMN, and SMN connectivity. Whole-brain linear regression between void volume and SLN (Fig. 5A) connectivity revealed significant clusters (p < 0.05) encompassing the left Posterior Insula (pINS), left Secondary Somatosensory Cortex (SII), and the Cerebellar Vermis (Fig. 5B). In addition, whole-brain linear regression between voided volume and DMN (Fig. 5C) connectivity revealed a significant cluster (p < 0.05) that encompassed the right Inferior Parietal Lobule (IPL) (Fig. 5D). Moreover, whole-brain linear regression between urinary urge and DMN connectivity revealed a significant cluster (p < 0.05) that encompassed the right Inferior Parietal Sulcus (IPS) and the SMA (Fig. 5E). Furthermore, whole-brain linear regression between void volume and SMN (Fig. 5F) connectivity revealed significant clusters (p < 0.05) that encompassed the ventromedial Prefrontal Cortex (vmPFC)/subgenual Anterior Cingulate Cortex (sgACC), right IPL, right Temporoparietal Junction (TPJ), and Left Dorsal Pons (Fig. 5G). Table 2 summarizes these clusters.

## Discussion

In the present study we implemented and demonstrated proof of concept of a natural bladder filling paradigm to assess the brain response across a comprehensive set of spatial scales (from local activity to network communication). This experimental procedure relied on natural diuresis instead of an invasive catheter-based urodynamic testing. This paradigm was successful in evoking a modest sense of urinary urge in a large portion of participants, who were designated as responders. The amount of urine collected upon complete voiding was linearly correlated with the perceived urinary urge (i.e. greater fullness of the bladder translated to greater urinary urge). In past brain-bladder studies, inferences about brain activity were obtained from contrasting different phases of bladder fullness (e.g., water infusion contrasted with post-void) but almost none of these studies have shown any associations between brain activity with participant-reported urinary urge or volume. Our study was able to overcome limitations of previous studies by demonstrating specific links of brain metrics with volume of urine voided (a proxy measure of bladder stimulus) and participant-reported urinary urge (resultant perception).

We measured brain function across different spatial scales. Local activity in the dACC and the SMA, as measured with fALFF in the slow-5 band, encoded the level of urinary urge. This was consistent with numerous catheter-based bladder infusion studies where the dACC and SMA are reliably activated during strong desire to void and full bladder^10,11,37,38^. The broader ACC regulates midbrain homeostatic centers^39^ and also supplies sympathetic outflow to the bladder^40^ whereas the SMA provides motor output^31,41^. The ACC and SMA provides signals that ultimately tightens the urethral sphincter and inhibits contraction of the detrusor muscles, allowing the bladder to be filled^8,40^. At the same time, we found that local activity in the sgACC, lateral OFC, and mPFC encoded the volume of urine voided. The mPFC may interact with arousal centers to play a role in shifting focus from ongoing activities unrelated to micturition to focusing on voiding in an appropriate environment (e.g., such as finding a bathroom)^42^. The OFC drives evaluative and goal-directed behavior^43^ and lower activation of the OFC has been linked to poor bladder control^6^.

Next, we examined circuits of a major hub related to micturition, the PAG. Neurons in the PAG display both tonic and phasic activity in response to both bladder storage and initiation of micturition^44^. These PAG neurons not only receive ascending input from bladder afferents, but also have rich bidirectional interconnections with higher-order regions, such as the prefrontal and limbic regions, which are essential for providing a wide range of conscious and behavioral responses (e.g., monitoring bladder fullness and voiding at an appropriate time)^2,8,45^. Consistent with the literature on the projections of the PAG^32,46^, our study found that during bladder storage, PAG connectivity to insular, frontal, cingulate, and motor subregions encoded the level of volume and urinary urge. These loci might be encoding different PAG signals—for instance, the DLPFC, being a cardinal node of the cognitive control networks, may be providing descending signals to the PAG^47^ to inhibit/not excite downstream micturition centers. At the same time, the SMA might be providing a motor output via the PAG to voluntarily engage the pelvic floor muscles to suppress urge and voiding until an appropriate circumstance arises^31,41^.

Finally, we examined large-scale networks in the brain (i.e. how a synchronized set of brain regions that form a network) interact with other brain regions in order to regulate bladder activity. Namely, we focused on the Default Mode (DMN), Sensorimotor (SMN), and Salience (SLN) networks. Several loci implicated in micturition belong to these networks—the mPFC belongs to the DMN, the SMA belongs to the SMN, and the ACC and the insula belongs to the SLN. We found several within- and cross-network connections that encoded urinary urge and volume. As one notable example, we found that the SLN connectivity to the pINS encoded volume; the pINS is a primary center for interoception where visceral signals from internal organs are processed (also known as visceroceptive cortex)^2,39^.

This natural bladder filling provides an easy-to-implement tool that does not depend on catheterization and hence can be scaled to large sample-size neuroimaging studies. In addition, it can be used to phenotype patients with urologic disorders, such as Urologic Chronic Pelvic Pain (UCPPS), which is a target clinical population to study for the MAPP network. Previous research from the MAPP network has shown that patients with hypersensitivity of the bladder can be classified into different clinical phenotypes^5,48^ In particular, patient bladder phenotypes can manifest as (a) neither painful filling or painful urgency, (b) either painful filling or urgency, and (c) both painful filling and urgency. Strikingly, patients in these categories demonstrate a stepwise worsening of both urologic and non-urologic symptoms, and have sex-specific clinical characteristics. Building on the results reported in this study for healthy individuals, the MAPP research network proposes to examine changes in brain function in both UCPPS compared to controls, as well as among the different clinical phenotypes of UCPPS. Furthermore, previous research from the MAPP network has shown that distributed resting brain connectivity pattern at baseline predicts the longitudinal trajectory of pain symptoms^49^; this prediction may be refined in future studies by better understanding the brain response to the bladder filling stimulus.

While the natural bladder filling paradigm described here has a number of advantages, it does not provide rapid control over bladder volume that is achievable in catheter-based urodynamic studies. Therefore, there are more experimental variables to consider in the natural bladder filling approach. For example, is a fixed volume of fluid provided for consumption, or do participants continue to consume fluid until a target degree of urinary urge is reached? In addition, despite our results showing no evidence for age-volume correlation, future versions of this protocol may want to administer an age-corrected drink volume due to the effects of age on bladder physiology. In this study, we report on a paradigm that was designed for use by the MAPP network to study UCPPS patients, many of whom report significant pain and urgency during natural bladder filling. During the design phase of this study, the 350 mL drink volume was chosen so as not to exceed tolerability for UCPPS patients, based on preliminary work assessing feasibility^4^. In future studies, the brain response to bladder filling will be compared between UCPPS patients and the controls described here. However, the 350 mL drink volume was not implemented with healthy individuals in mind and evidently was not sufficient to evoke a large urge for all healthy participants, hence necessitating a subdivision of responders and non-responders. In addition, the mean evoked urge was only ~ 3 out of 10 for the responder group, which is much lower than many previously published urodynamic studies that drive the bladder to full capacity to maximize urge. Therefore, our study only offers a proof of concept of an ecologically relevant task and needs further validation with various samples and higher drink volume in order to (1) maximize the number of responders, (2) to maximize urinary urge, or (3) to maximize the brain response. Another limitation of our subdivision into responder and non-responder is a lack of direct comparison of whole-brain regressions between the two groups, as the urge values for non-responders were centered around zero. Furthermore, unlike urodynamic studies, bladder capacity was not measured, so the percentage of capacity that volume represents is not known—however, we argue that bladder capacity is not relevant here as urge is a function of the volume present in the bladder (Fig. 2B). While we acknowledge these limitations of the natural bladder filling approach, this report nonetheless demonstrates that the natural bladder filling paradigm does elicit a robust brain response at multiple spatial scales even in healthy individuals, laying the necessary groundwork for analytic approaches in future studies to examine patients with underlying urologic disorders.
